# Apolipoprotein E Gene Polymorphism and Risk for Coronary Heart Disease in the Chinese Population: A Meta-Analysis of 61 Studies Including 6634 Cases and 6393 Controls

**DOI:** 10.1371/journal.pone.0095463

**Published:** 2014-04-22

**Authors:** Ming-duo Zhang, Wei Gu, Shi-bin Qiao, En-jun Zhu, Quan-ming Zhao, Shu-zheng Lv

**Affiliations:** 1 Department of Cardiology, Beijing Anzhen Hospital, Capital Medical University, Beijing, China; 2 Beijing Institute of Heart, Lung and Blood Vessel Diseases, Beijing, China; 3 Department of Cardiac Surgery, Beijing Anzhen Hospital, Capital Medical University, Beijing, China; 4 Department of Cardiology, The Fourth Affiliated Hospital of Anhui Medical University, Anhui, China; 5 Department of Cardiology, Rizhao People’s Hospital, Shandong, China; University of Tampere, Finland

## Abstract

**Background:**

Numerous studies have evaluated the association between the apolipoprotein E (apoE) gene polymorphisms in coronary heart disease (CHD). However, the results remain uncertain. We carried out a meta-analysis to derive a more comprehensive estimation of the association in Chinese population.

**Methods:**

Case-control studies in Chinese and English publications were identified by searching databases of PubMed, EMBASE, Web of Science, CNKI, CBM, Wanfang, VIP and hand searching of relevant journals and the reference lists of retrieved articles. Odds ratio (OR) and 95% confidence interval (CI) were applied to assess the strength of the associations. Subgroup analysis and sensitivity analysis were performed to explore the between-study heterogeneity.

**Results:**

We finally identified 61 relevant studies which comprised 6634 case-patients and 6393 controls. The pooled OR for ε4 carriers was 96% higher than the ε3/3 genotype for CHD (OR, 1.96; 95% CI, 1.70 to 2.24; P<0.001). However, there was no evidence of statistically significant association between ε2 carriers and risk of CHD (OR, 1.02; 95% CI, 0.91 to 1.13; P = 0.729). In the subgroup analysis, different endpoints may partially account for the heterogeneity. No publication bias was found.

**Conclusions:**

Our meta-analysis suggests that the apoE ε4 allele may be a risk factor for CHD in the Chinese population, however, ε2 allele has no significant association.

## Introduction

Coronary heart disease (CHD) is one of the leading causes of death and disability around the world [1]. CHD is generally regarded as a multifactorial disorder that is associated with genetic and environmental factors [2], [3], [4]. Currently, many candidate genes for CHD have been extensively investigated especially some encoded genes which is linked to metabolic abnormalities of lipoproteins [5].

Mounting evidence suggested that Apolipoprotein E (apoE) is one of the candidate [6], [7]. ApoE is a receptor-binding ligand protein of liver, which can mediate the metabolism of cholesterol and triglyceride by clearance of chylomicron and remnants of very-low-density lipoprotein(VLDL) cholesterol from plasma [8]. ApoE also influences the metabolism of the lipoproteins to which it is associated, independently of its interaction with its receptors. Three common variant alleles (ε2, ε3 and ε4) of *apoE* gene generates 6 different genotypes (ε2/2, ε2/3, ε2/4, ε3/3, ε3/4, and ε4/4). These alleles have variant frequencies across the populations [9]. The three corresponding encoded isoforms: E2, E3 and E4 have different functional properties [10]. E2 with a very low affinity for the low-density lipoprotein (LDL) receptor has a delayed clearance of VLDL and chylomicron remnants, on the other hand, E4 is characterized by a preferential binding to VLDL. Many studies assessing the role of apoE genetics on plasma lipids have indicated that the presence of E4 is associated with elevations in LDL cholesterol, while E2 is associated with decreased levels of LDL cholesterol [11]. Since researchers [8] first described the effects of apoE polymorphism on dysbetalipoproteinemia, a considerable amount of studies have explored the association between *apoE* gene and CHD risk in the general population [12]. ApoE polymorphism is believed to confer substantial effect on CHD risk. Of note, differences features such as ethnicity, sources of controls among studies have led to discrepancy in estimating the true effect of apoE genotypes on CHD risk.

In 2004, a meta-analysis [13] reported that compared with carriers of the apoE ε3/3 genotype, carriers of the apoE ε4 allele had a significant increased risk for CHD (OR, 1.30; 95% CI, 1.18 to 1.43), whereas the ε2 allele had no effect (OR, 0.93; 95% CI, 0.83 to 1.05). But few studies included in this meta-analysis was from Chinese. Another meta-analysis published in 2007 by Bennet et al. [12], compared with ε3/3 individuals, ε2 carriers have a 20% reduced risk of CHD whereas ε4 carriers have only a slightly increased risk. Also, little data was related to Chinese. The difference of genetic background between Caucasian and Chinese may lead to different results. Moreover, the results of studies published for apoE polymorphism in Chinese remains uncertain. Some studies have indicated notably significant associations, while others have shown null association. Therefore, we performed a carefully designed meta-analysis to clarify the association between *apoE* gene polymorphism and CHD risk in Chinese populations.

## Methods

We followed the Preferred Reporting Items for Systematic Reviews and Meta-Analyses (PRISMA ) statement [14] to report the present meta-analysis. A PRISMA checklist is shown in Table S3.

### Identification and Search of Relevant Studies

To search for all the studies that examined the association of apoE polymorphisms with CHD in Chinese, we conducted a comprehensive literature search of PubMed, EMBASE, Web of Science, CNKI(China Nation Knowledge Infrastructure Platform), CBM (China Biological Medicine Database), Wanfang, and VIP databases (up to October 2013), using the following MeSH terms and keywords: ‘Apolipoprotein E’, ‘apoE’, ‘APOE’, ‘polymorphism’, ‘atherosclerosis, ‘coronary heart disease or CHD’, ‘coronary artery disease or CAD’, ‘ischemic heart disease or IHD’, ‘myocardial infarction or MI’ and ‘Chinese or China or Taiwanese or Taiwan’. All eligible studies were performed in human. We also screened all the reference lists of retrieved articles and review articles (including systematic reviews and meta-analyses). We also retrieved additional studies by hand searching of relevant journals and by correspondence with authors of included studies. If there were multiple publications from the same study group, to prevent data duplication, the most complete and recent results were kept. We exclusively included studies published in Chinese or English.

Studies satisfying the following predefined criteria were identified: (i) retrospective case–control studies using either a hospital-based or a population-based design(family-based study design was excluded); (ii) evaluation of *apoE* gene polymorphism with the risk of CHD in Chinese population; (iii) definition of CHD endpoints included myocardial infarction, coronary stenosis on coronary angiography (≥50% in at least 1 of the 3 major coronary arteries); (iv) without consanguinity between cases and controls; (v) sufficient information was supplied for estimating the odds ratio (OR) and its corresponding 95% confidence interval (CI) between cases and controls.

### Data Extraction

To minimize the selection bias, two authors (Ming-duo Zhang and Wei Gu) independently reviewed and extracted the data needed. Disagreements were resolved through discussion between the authors to achieve a consensus. From each study the following information was abstracted: first author, publication year, resident region of population studied, racial background, diagnostic criteria, the number of sample in both the case and control groups, number of cases and controls, the characteristics of the case group (sex, age and endpoint) and the control group (sex, age and source of control) within each study, distribution of genotypes and alleles in both case and control groups.

### Statistical Analysis

Case–control studies were used, OR and 95% CI were applied to assess the strength of the association of *apoE* gene polymorphisms with CHD, which was calculated according to Woolf method [15]. Heterogeneity among studies was calculated using the *χ* ^2^-based Cochran's Q-statistic test [16] (P<0.10 was considered statistically significant heterogeneity), the inconsistency index *I* ^2^ statistic was also calculated to observe between-study variability that was due to heterogeneity rather than chance [17]. This statistic, which was documented by percentage, yields result ranging from 0 to 100% (*I* ^2^ = 0–25%, no heterogeneity; *I* ^2^ = 25–50%, moderate heterogeneity; *I* ^2^ = 50–75%, large heterogeneity; *I* ^2^ = 75–100%, extreme heterogeneity) [17]. A fixed-effects model using the Mantel and Haenszel method was used in the absence of between-study heterogeneity, and a random effects model using the method of DerSimonian & Laird was used to investigate variation both from in-study and between-study. Either a random-effects model or fixed-effects model was used to combine pooled effect estimates in the presence or absence of heterogeneity, respectively. The significance of the pooled OR was determined by the Z test (*P*<0.05 was considered significant). As apoE ε3/3 genotype is the most common genotype in population with a frequency of about 67%, it is widely considered as the “wild-type” genotype [18]. Thus, Individuals with the ε3/3 genotype were designated as the reference group in the present study. For separate analyses, ε2 carriers included patients with the ε2/2 or ε2/3 genotype, ε4 carriers included patients with the ε3/4 or ε4/4 genotype. ε2 and ε4 carriers were compared with the ε3/3 genotype respectively. Separate analyses were conducted for each genotype (in the following order: ε2/2, ε2/3, ε2/4, ε3/3, ε3/4, and ε4/4) and for ε2 and ε4 carrier status. If unexpected heterogeneity was present, we undertook subgroup analysis to explore the potential sources of between-study heterogeneity in included studies. A variety of prespecified variables such as ethnicity, control sources, endpoint, assay type and sample size of published studies were included in our analysis [13]. Each subgroup had at least two independent studies.

Sensitivity analysis was performed to examine whether the findings in the meta-analysis were robust. One way was conducted by sequential removal an individual study each time and then examined whether any of the ORs can bias the results. Another method was to identify if the overall significance of the estimate is altered when we excluded studies with deviation from the Hardy-Weinberg Equilibrium (HWE) among controls. Publication bias was investigated by a Begg modified funnel plot, in which the standard error of the log (OR) of each study was plotted against its OR. An asymmetric plot suggested possible publication bias. Funnel-plot asymmetry was assessed by the method of the Begg adjusted rank correlation test [19] and Egger regression test [20]. *P*<0.05 was considered statistically significant. All statistical analyses were performed by using STATA statistical version 10.0 (Stata Corporation, College Station, Texas, USA). All *P*-values were two-sided.

## Results

### Studies Included in the Meta-analysis

**Figure 1:**
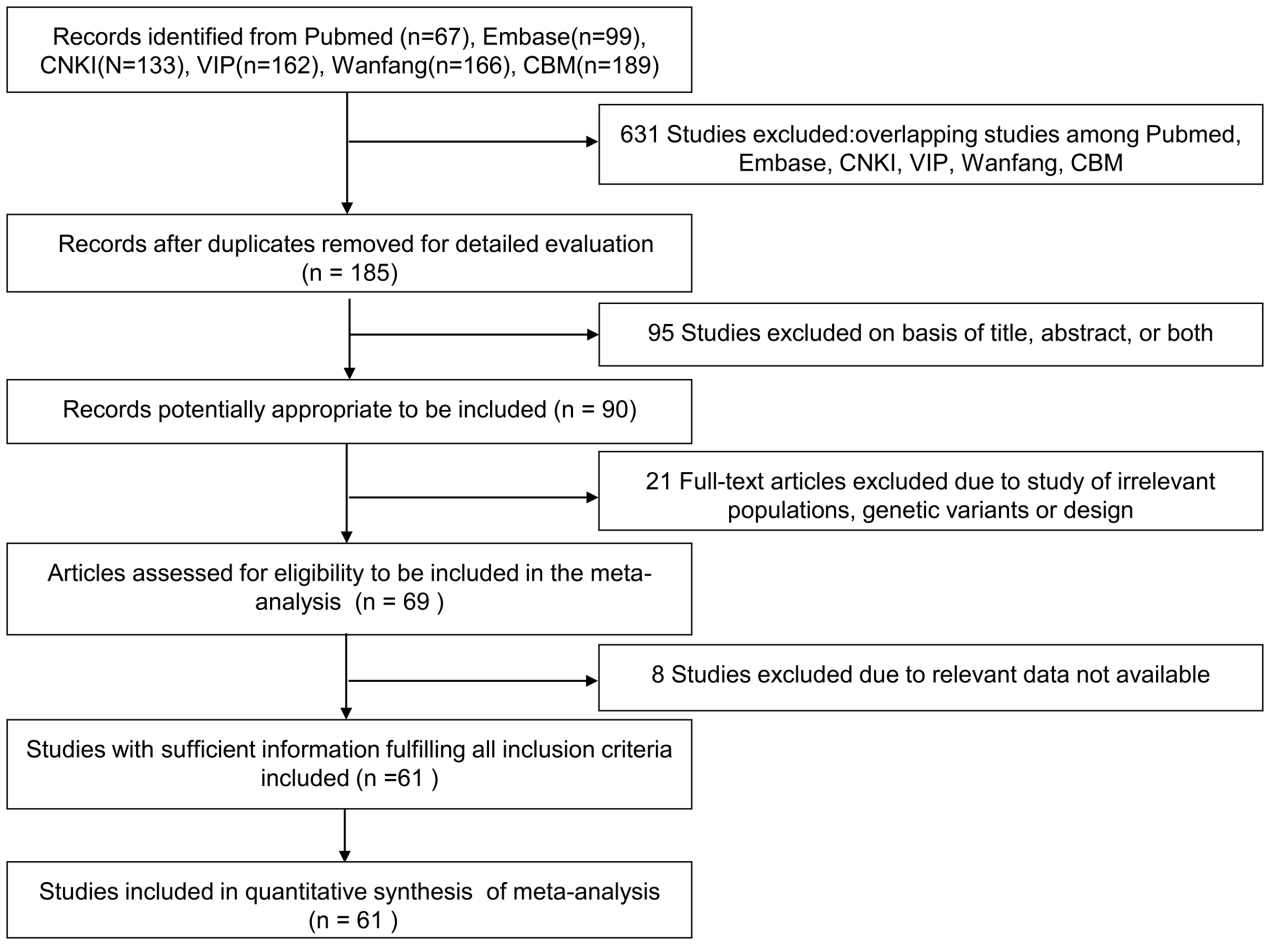
The flowchart of selection of studies.

After a comprehensive literature search applying our inclusion criteria, 61 relevant studies which comprised 6634 case-patients and 6393 controls from 816 potentially relevant articles were identified in the final analysis (Figure 1).

Fifty-five studies involved Han Chinese, whereas the other 6 studies were performed in non-Han Chinese. Of all the 61 studies, controls of 23 studies came from general population and the rest were hospital-based ones (Table S1). The controls of 46 studies met HWE of genotype distributions (Table S2). The overall genotype frequencies among people without CHD were 0.012 for ε2/2, 0.128 for ε2/3, 0.022 for ε2/4, 0.708 for ε3/3, 0.121 for ε3/4, and 0.008 for ε4/4. The overall allele frequencies were 0.087 for ε2, 0.813 for ε3, and 0.092 for ε4. These frequencies varied among studies. However, the ε3 allele still was the most common, and ε3/3 consistently had the highest frequency among studies. The detailed characteristics of the included studies are presented in Table S1 and S2.

### Main Meta-results

**Figure 2:**
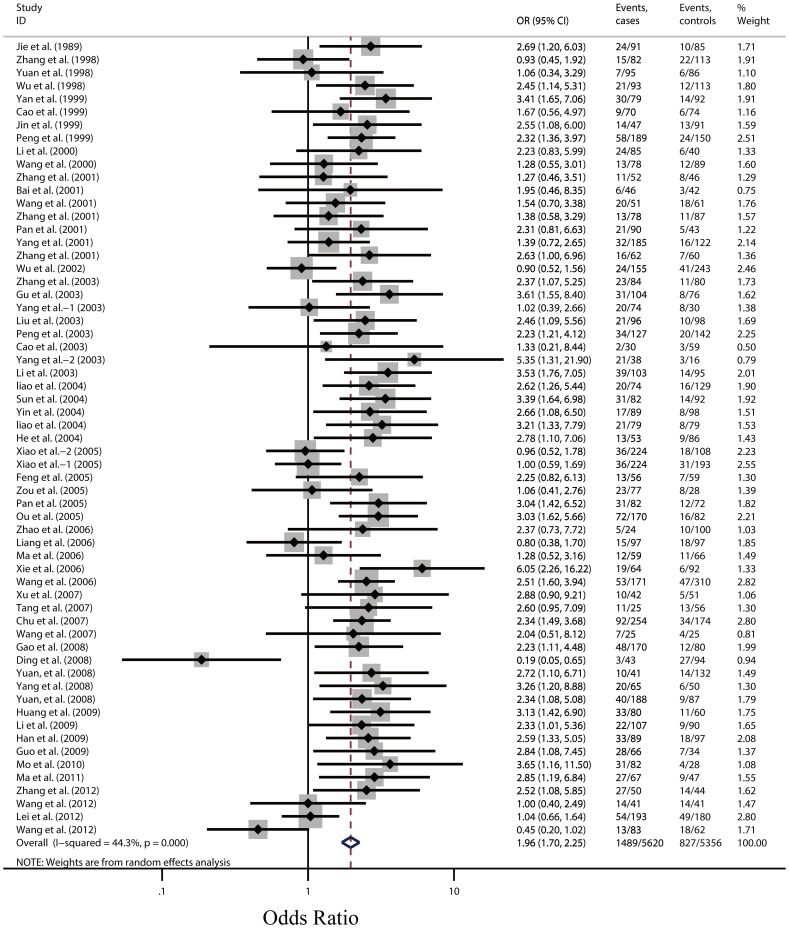
Odds ratios for coronary heart disease in ε4 carriers versus population with the ε3/3 genotype of all 61 studies. Size of the squares is proportional to the weight of the odds ratios; black circular dots indicate the odds ratios; horizontal lines represent the 95% CI. Dark hollow diamonds show the pooled estimates from the random-effects models (with 95% CI) and the fixed-effects model (with 95% CI).

**Figure 3:**
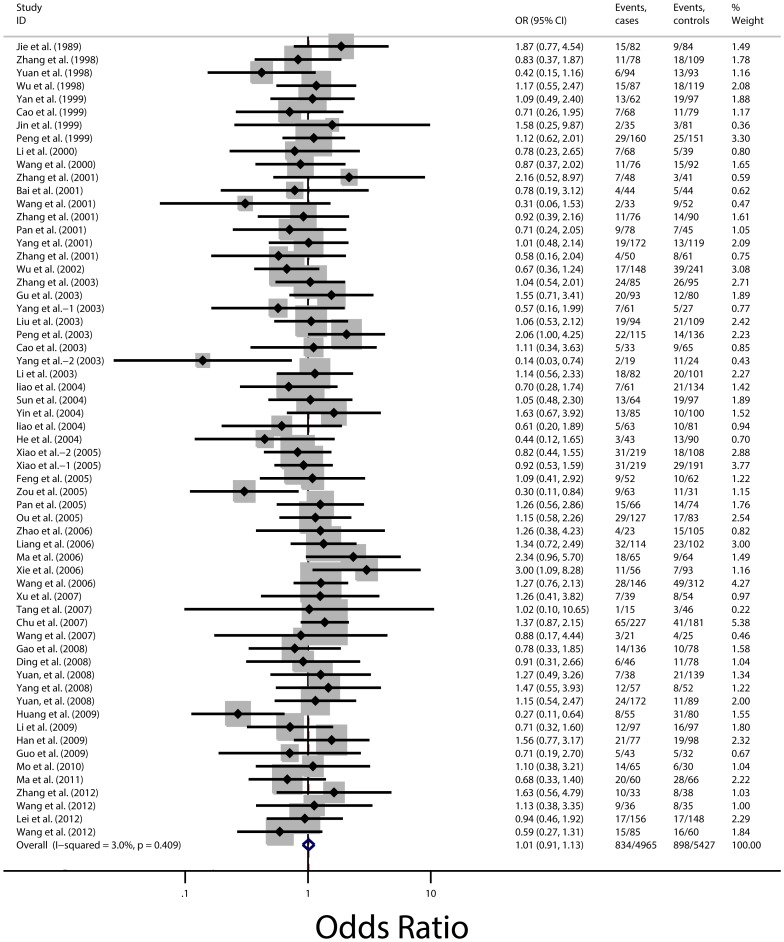
Odds ratios for coronary heart disease in ε2 carriers versus population with the ε3/3 genotype of all 61 studies. Size of the squares is proportional to the weight of the odds ratios; black circular dots indicate the odds ratios; horizontal lines represent the 95% CI. Dark hollow diamonds show the pooled estimates from the random-effects models (with 95% CI) and the fixed-effects model (with 95% CI).

**Figure 4:**
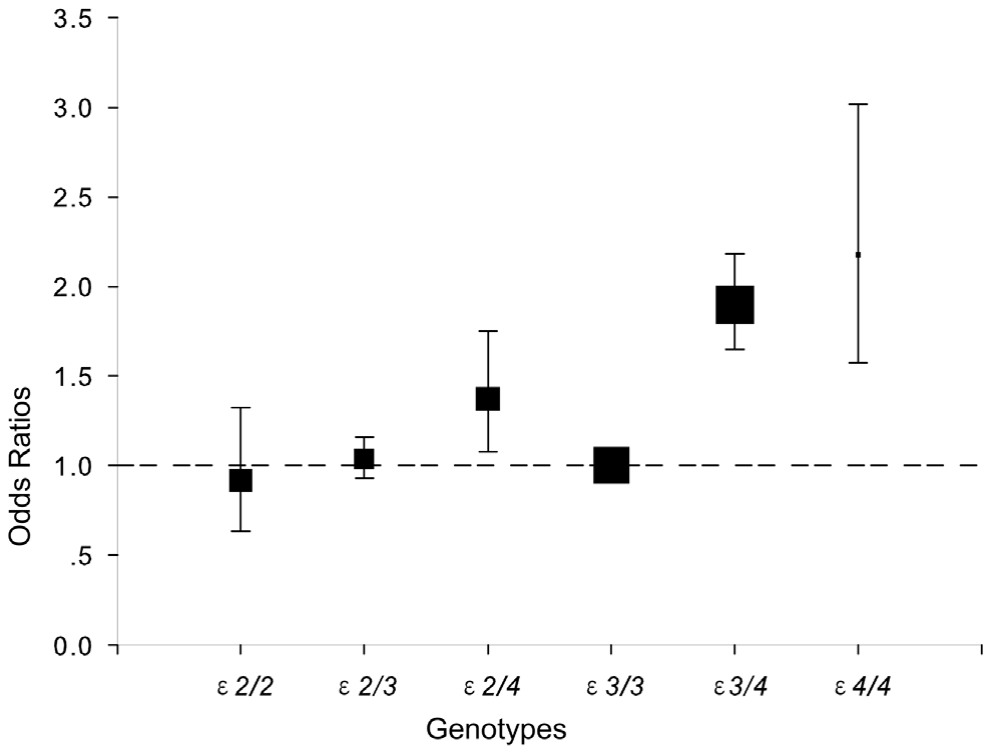
The comparison of ORs between ε3/3 and all the other genotypes for coronary heart disease, based on 61 studies. Size of data symbols is proportional to the inverse of the variance of odds ratios (ε3/3 is displayed with forced fixed size) and error bars represent 95% confidence intervals (CIs).

The pooled ORs for CHD in ε2 carriers and ε4 carriers with the ε3/3 genotype as the reference group are shown in Figure 2 and 3. Figure 2 reveals the combined ORs in ε4 carriers had a 96% higher risk than the ε3/3 genotype for CHD (OR, 1.96; 95% CI, 1.70 to 2.24; P<0.001), we found significant between-study heterogeneity and the random effect model was used. However, there was no evidence of statistically significant association between ε2 carriers and CHD risk (OR, 1.02; 95% CI, 0.91 to 1.13; P = 0.729), no evidence of heterogeneity were found, hence, fixed-effects model was employed, as shown in Figure 3. The pooled estimates for the comparison between ε3/3 genotype and each of the other genotypes (ε2/2, ε2/3, ε2/4, ε3/4, and ε4/4) displayed different results. People with the genotypes of ε2/4, ε3/4 and ε4/4 had significantly higher risk for CHD (ORs, 1.37; 95% CI, 1.08 to 1.75; 1.90; 1.65 to 2.18 and 2.18; 1.57 to 3.02, respectively) than those with the genotype of ε3/3, whereas the differences between CHD risk and ε2/2 or ε2/3 genotype were not significant (ORs, 0.92; 95% CI, 0.64 to 1.32 and 1.04; 0.93 to 1.16, respectively) (Figure 4). No evidence of heterogeneity were also found, hence, fixed-effects model was employed in these comparisons. The summary estimates of the ORs examining the association between the given alleles and CHD risk compared with the ε3 allele showed significantly higher for the ε4 allele (OR, 1.78; 95% CI, 1.57 to 2.01) but not for the ε2 allele (OR, 0.99; 95% CI, 0.88 to 1.10).

### Subgroup Analysis

We performed Subgroup analysis according to the prespecified variables. In general, although people with the ε4 carriers had considerably increased risk for CHD in both overall and separate analysis, heterogeneity was found in most subgroups in the comparison of ε4 carriers with the reference genotype of ε3/3 (Table 1). Different CHD endpoints may partially account for the heterogeneity. The increased risk for CHD was significant for stenosis associated with the comparison of ε4 carriers with the ε3/3 genotype (OR: 1.99, 95% CI: 1.71 to 2.32, P<0.001) with moderate heterogeneity across studies (P_heterogeneity_ <0.001, I^2^ = 45.50%). However, there was a 95% increased risk for mixed endpoint (Stenosis or MI) with no evidence of significant heterogeneity across studies (P_heterogeneity_ = 0.239, I^2^ = 27.4%). In the subgroup analysis by ethnicity, all studies were divided into two groups: Han Chinese and non-Han Chinese. Han Chinese included more than 90% of studies (55 out of 61), including 6097 cases and 5785 controls, non-Han Chinese comprised 6 studies. Both subgroups had a markedly increased risk (OR, 1.98, 95% CI, 1.71 to 2.29, P<0.001 for Han Chinese and OR, 1.82, 95% CI, 1.11 to 2.96, P = 0.017 for non-Han Chinese; respectively) for CHD in the comparison of ε4 carriers with the ε3/3 genotype, with moderate heterogeneity across studies. For the subgroup analysis based on different sources of controls, people from hospital had a 109% increased risk for CHD, whereas a 73% increased risk was found in those from general population. Both two kinds of sources had moderate heterogeneity. Assay type are different across studies, PCR-based methods are used in 57 studies and Sequencing analysis is used in the other 4 studies. The increased risk for CHD was most evident for the group of Sequencing analysis with large heterogeneity among studies(OR: 2.36, 95% CI:1.07 to 5.21, P_heterogeneity_ = 0.009, I^2^ = 74%). Analysis by grouping the studies according to the sample size of published studies showed that smaller studies yielded larger ORs and corresponding CIs in the comparison of ε4 carriers with the ε3/3 genotype (OR: 1.98, 95% CI: 1.70 to 2.29, P<0.001 for sample size less than 100 and OR: 1.87, 95% CI: 1.64 to 2.13, P<0.001 for for sample size at least 100; respectively). In contrast, the differences relating to the comparison of ε2 carriers with the ε3/3 genotype for CHD were not significant in both overall and separate analysis, except for the subgroup of non-Han Chinese with a notably reduced risk (OR: 0.53, 95% CI: 0.29 to 0.96, P = 0.037). Both overall and almost all subgroups analyses displayed no evidence of heterogeneity in those comparisons.

**Table 1:** Odds Ratios of the Apo E Gene Polymorphisms and the Risk for Coronary Heart Disease, Results of Subgroup Analysis.

		e2 carriers*			e4 carriers*		
Subgroup variables	Studies(cases/controls)†	OR(95% CI)	P*_heterogeneity_* ‡	I^2^(%)	OR(95% CI)	P*_heterogeneity_* ‡	I^2^(%)
Overall	61(6634/6393)	1.01(0.91–1.13)	0.409	3.00	1.96(1.70–2.25)	0.000	44.30
**Ethnicity**							
Han Chinese	55(6097/5785)	1.06(0.95–1.18)	0.828	0.00	1.98(1.71–2.29)	0.001	42.60
Non-Han Chinese	6(537/608)	0.53(0.29–0.96)	0.082	48.80	1.82(1.11–2.96)	0.037	57.70
**Control sources**							
Population-based	23(2302/2351)	0.88(0.72–1.07)	0.509	0.00	1.73(1.38–2.18)	0.031	38.80
Hospital-based	38(4332/4042)	1.08(0.95–1.23)	0.436	1.90	2.09(1.77–2.47)	0.004	42.00
**Endpoint**							
Stenosis	52(5931/5467)	1.06(0.95–1.19)	0.661	0.00	1.99(1.71–2.32)	0.000	45.50
MI	4(336/561)	0.62(0.38–1.03)	0.563	0.00	1.64(0.93–2.86)	0.093	53.20
Stenosis or MI	5(367/365)	0.58(0.29–1.18)	0.161	39.00	1.95(1.15–3.33)	0.239	27.40
**Assay type**							
PCR-based	57(6216/5970)	0.98(0.88–1.10)	0.554	0.00	1.94(1.68–2.23)	0.001	41.60
Sequencing analysis	4(418/423)	1.30(0.91–1.87)	0.113	49.70	2.36(1.07–5.21)	0.009	74.00
**Smple size**							
≥100	37(2626/2924)	1.11(0.96–1.27)	0.750	0.00	1.87(1.64–2.13)	0.003	49.70
<100	24(4008/3469)	0.87(0.74–1.03)	0.307	9.40	1.98(1.70–2.29)	0.005	41.50

OR, Odds Ratios; CHD, coronary heart disease; CI, confidence interval; MI, myocardial infarction; PCR, polymerase chain reaction.

*ε2 carriers comprises ε2/2 and ε2/3; ε4 carriers comprises ε3/4 and ε4/4.

† The Arabic numerals in parentheses represent the total number of cases and controls.

‡ The significance level of the statistics P*_heterogeneity_* is 0.1.

### Sensitivity Analysis

The influential analysis showed that no particular study affected the overall significance of the pooled estimates. After removing each study and recalculating the ORs, overall estimates as well as their significance remained nearly unchanged in both the ε2 carriers comparison and the ε4 carriers comparison with the ε3/3 genotype as can be seen from Figure S1. The pooled estimates were also not materially altered when we excluded studies which deviated from HWE among controls of ε4 carriers (OR: 1.95, 95% CI: 1.66 to 2.29, P_heterogeneity_ = 0.001) and ε2 carriers (OR: 1.06, 95% CI: 0.93 to 1.20, P_heterogeneity_ = 0.458).

### Publication Bias

The shape of the funnel plot does not display any evidence of apparent asymmetry for the ε4 carriers with CHD risk, furthermore, the formal tests also show no evidence of substantial publication bias (P = 0.608 for the Begg test; P = 0.605 for the Egger test). Similarly, neither funnel plots nor formal tests show publication bias for the ε2 carriers (P>0.05 for both Begg and Egger tests) as can be seen from Figure S2.

## Discussion

To our knowledge, this present meta-analysis of 61 studies, with a total of 6 634 CHD cases and 6 393 controls, provides the most comprehensive assessment of the association between *apoE* gene polymorphisms and CHD in Chinese population. Our meta-analysis indicates that ε4 carriers conferred a significant 96% higher risk than the ε3/3 genotype for CHD. However, we found no evidence of statistically significant association between the ε2 carriers and CHD risk. For the comparison of genotypes separately, people with the genotypes of ε2/4, ε3/4 and ε4/4 had significantly higher risk for CHD than ones with the genotype of ε3/3, whereas the ε2/2 and ε2/3 genotypes were not significant.

The detailed mechanism by which ε4 allele carriers might confer adverse lipid profiles is partially understood. Several studies have examined apoE with lipid metabolism, especially LDL cholesterol levels, and CHD risk and the mechanism of effect has been discussed in these papers [8], [21], [22]. Evidence shows that ApoE polymorphisms may account for 2 to 11% of the total variance present in the serum or plasma cholesterol levels of apparently healthy Caucasians [23], [24]. Plasma total cholesterol and LDL cholesterol levels tended to be higher among individuals of ε4 carriers as compared with ε3/3 ones.

ApoE4 is also associated with increased ApoB and cholesterol levels and decreased apoE levels [6]. In addition to cardiovascular disease, *apoE* gene polymorphisms were associated with many pathophysiological conditions, including Alzheimer’s disease (AD), diabetes,Parkinson’s disease, renal disease and stroke [6], [11], [25], [26], [27]. The normal role of three major isoforms of apoE is related to their receptor affinity [28]. The most common is apoE3, which is found in maintenance of normal activities. However, the apoE4 is seen as a ‘thrifty’ gene and the important functions of apoE4 are to increase cholesterol production in the liver [6]. Therefore, apoE4 tends to reduce the level of high-density lipoprotein (HDL) and increase the level of LDL cholesterol in the high-fat intake population [29]. Many other factors such as inflammation [30], [31], immunity [32] and oxidative status [33] might also exert interactive influences on the lipid profiles. Meta-analysis performed by Bennet et al. [12]displayed that people with the ε2/ε2 genotype had about 30% lower mean LDL-C values than those with the ε4/ε4 genotype. There were approximately linear relationships of apoE genotypes with LDL-C and with coronary risk. However, in our meta-analysis, little studies included the lipid profile of participants, future studies with blood lipids characteristics were needed to confirm those findings in Chinese population. The detailed mechanism underlying the association of apoE polymorphism with CHD risk is not completely addressed and our findings should stimulate further investigation.

The results of our study are in general agreement with those performed by Song et al. [13], but contrast with those performed by Bennet et al. [12]. Most persons included in the two meta-analyses are Caucasian, whereas little data is focus on Chinese. Ethnicity may contribute to the difference. The results of another meta-analysis [34] which tried to explore the association between ε4 allele and CHD risk in Chinese were consist with our current study. However, these investigators neither investigated the relation between ε2 allele and CHD risk nor performed subgroup or meta-regression analysis to further explore the potential sources of between-study heterogeneity. Our study included more relevant articles and we performed a wide range of predefined subgroup analyses.

We should emphasize that heterogeneity is an important concern in meta-analysis. We performed comprehensive subgroup analyses and sensitivity analyses to further explore the potential sources of between-study heterogeneity. The subgroup analyses included multiple covariates, comprising ethnicity, control sources, genotyping methods, sample size and end point. Especially, different ethnicity and endpoints may partly account for the heterogeneity. The findings of our meta-analysis indicated that non-Han Chinese had a decreased risk for CHD in the comparison of ε2 carriers with ε3/3 genotype, but not for Han Chinese. However, the majority of subjects were limited to Han Chinese, so the results for non Han Chinese might be unreliable. For different endpoints, although with evident heterogeneity, ε4 carriers was strongly associated with coronary stenosis. Populations of MI and mixed endpoint had the same results but with little heterogeneity. Song et al. [13] also found that ε4 carriers was associated with CHD death which was consist with our study. Hospital-based studies yielded a more significant association than population-based ones. Generally, population-based studies offers more advantages over hospital-based design in minimizing false-positive findings related to selection bias [35]. hospital-based controls may have other diseases and also been given the corresponding drugs which exerted a confounding effect on the risk for CHD. Thus, we can not completely exclude the possibility that a true genetic effect was overestimated. The results of studies with population-based controls may be more reliable, and the results of hospital-based studies should be explained with caution.

Analysis by grouping the studies according to the sample size of published studies showed that smaller studies yielded larger ORs and corresponding CIs in the comparison of ε4 carriers with the ε3/3 genotype. These results displayed that small study-related bias was very likely because of smaller sample size. However, the risk associations between ε4 carriers and ε3/3 genotype were broadly similar in subgroups divided by the other covariates. Equally, ε2 carriers had the same results. Heterogeneity can not totally rule out in our study, so we dealt with this concern also by using multiple sensitivity analyses to identify the robust of the findings. The pooled estimates were not materially altered when we excluded studies which deviated from HWE among controls. Deviation from HWE among controls may suggest potential selection bias of controls or genotyping errors and tend to overestimate the chance of a false-positive association [36]. However, the pooled estimates were not materially altered when we excluded studies which deviated from HWE among controls, these studies were still included in our analysis. The influential analysis displayed that no study affected the overall significance of the pooled estimates.

Publication bias may introduce false positive in a meta-analysis [20]. So every effort should be made to avoid possible bias. In order to avoid this bias, we included both English and non-English articles. Studies whose controls deviates from the HWE were all properly assessed. Begg and Egger test for detecting publication bias were performed and no evident bias was found.

The present meta-analysis should be interpreted with caution, several limitations merit consideration. First, in this study, we only focused on *apoE* gene polymorphisms and were not able to evaluate other genes responsible for CHD. Some genes such as ApoB gene may have interaction with *apoE* gene by gene-gene effect for CHD. Second, publication bias can not entirely excluded, like all other meta analyses, might potential distort the conclusion, since all the included studies in our meta-analysis are all from either English or Chinese journals and many small size studies were included. However, since the ε2 allele has a very low frequency (0.087) in the Chinese population, the lack of association may be due to the small sample sizes.Third, we are unable to obtain enough data from original studies to adjust for potential confounding factors by performing additional subgroup analysis. These factors such as age, sex, smoking and alcohol consumption which have been regarded as effective modulators for the development of CHD.

In summary, our meta-analysis indicates that ε4 allele has an increased risk for CHD in Chinese, but the ε2 allele has null association except the subgroup of non-Han Chinese. More large-scale and incorporated with various covariates studies should be performed to further elucidate the association between the *apoE* gene polymorphisms and CHD in the Chinese populations.

## Supporting Information

Figure S1 **Influence analysis of people with ε2 carriers (A) and ε4 carriers (B) versus those with the ε3/3 genotype for the risk of coronary heart disease.** Open circle indicates the pooled ORs, horizontal lines represent the 95% CIs, given named study is omitted.
(TIF)

Figure S2 **Begg's funnel plot for comparison of ε2 carriers (A) and ε4 carriers (B) with ε3/3 genotype for the risk of coronary heart disease.** Size of the open circles is proportional to the weight of studies.
(TIF)

Table S1 **Baseline characteristics of the studies included in the meta-analysis.**
(DOCX)

Table S2 **Sample size, the distribution of Apo E genotypes, alleles frequencies, P values and** ***χ*** **^2^ values of HWE.**
(DOCX)

Table S3 **PRISMA 2009 checklist.**
(DOCX)
