# Supplementary figures and images for: Apolipoprotein E Gene Polymorphism and Risk for Coronary Heart Disease in the Chinese Population: A Meta-Analysis of 61 Studies Including 6634 Cases and 6393 Controls
Source: PLoS One. 2014 Apr 22;9(4):e95463. doi: 10.1371/journal.pone.0095463 (PMC3995769; doi:10.1371/journal.pone.0095463)

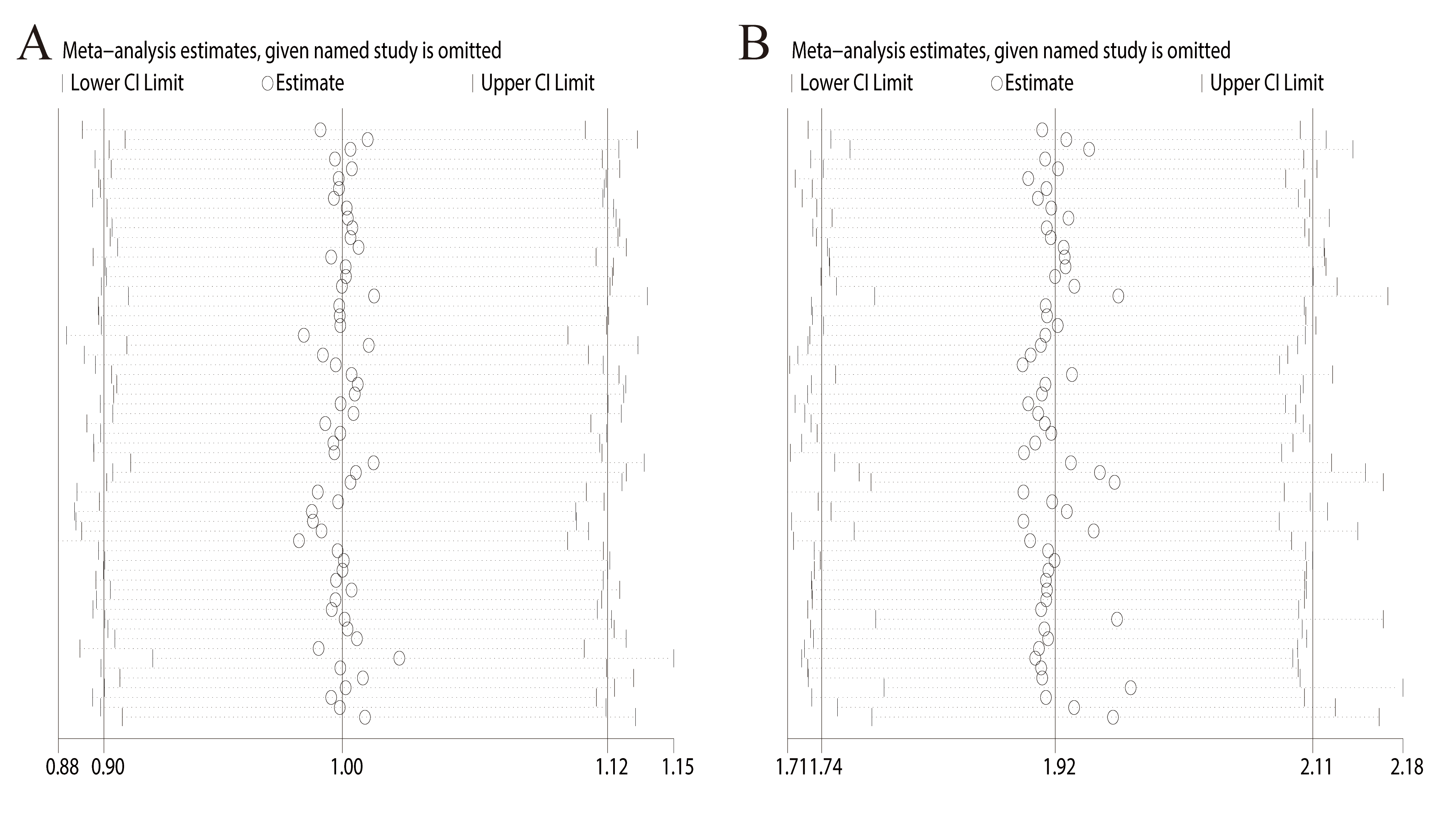

Supplement: Figure S1 — Influence analysis of people with ε2 carriers (A) and ε4 carriers (B) versus those with the ε3/3 genotype for the risk of coronary heart disease. Open circle indicates the pooled ORs, horizontal lines represent the 95% CIs, given named study is omitted. (TIF) [file pone.0095463.s001.tif]

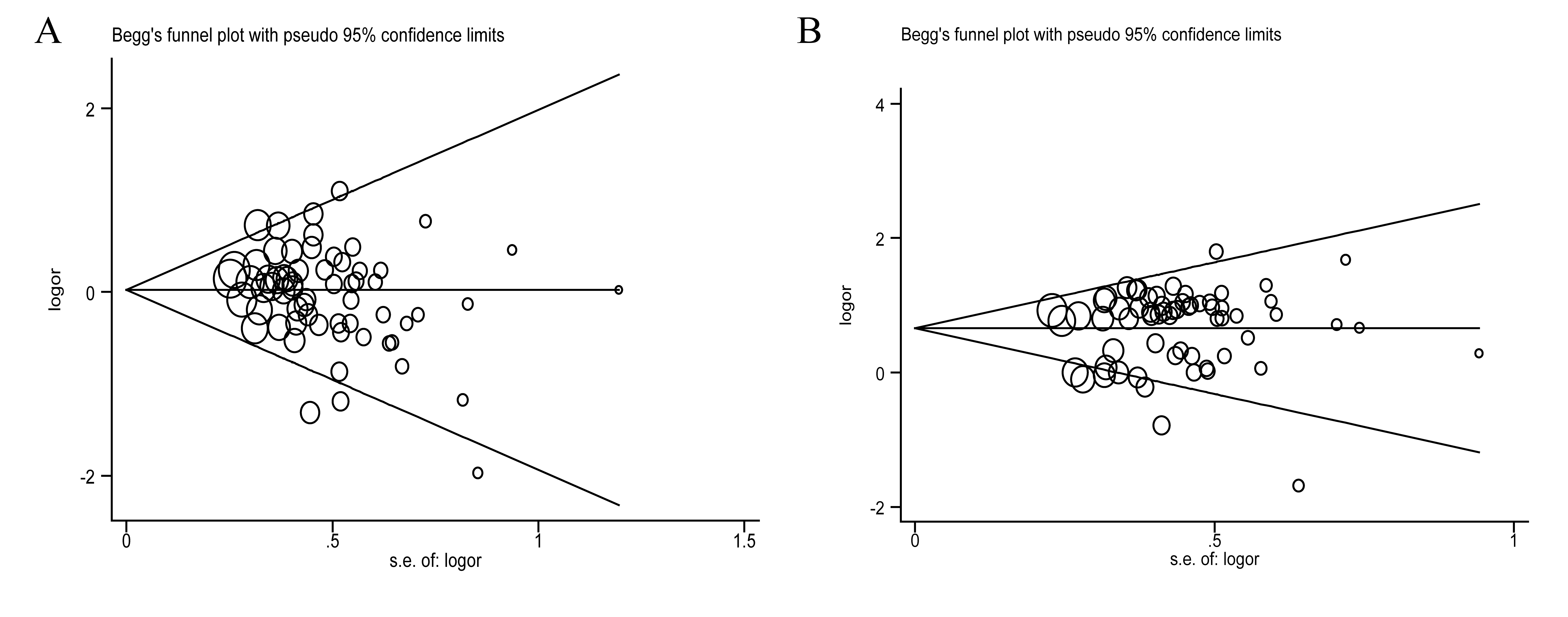

Supplement: Figure S2 — Begg's funnel plot for comparison of ε2 carriers (A) and ε4 carriers (B) with ε3/3 genotype for the risk of coronary heart disease. Size of the open circles is proportional to the weight of studies. (TIF) [file pone.0095463.s002.tif]
