# Supplementary material for: Apolipoprotein E Gene Polymorphism and Risk for Coronary Heart Disease in the Chinese Population: A Meta-Analysis of 61 Studies Including 6634 Cases and 6393 Controls
Source: PLoS One. 2014 Apr 22;9(4):e95463. doi: 10.1371/journal.pone.0095463 (PMC3995769; doi:10.1371/journal.pone.0095463)
Supplement: Table S2 — Sample size, the distribution of Apo E genotypes, alleles frequencies, P values and χ 2 values of HWE. (DOCX) [file pone.0095463.s004.docx]

**Table S2. Sample size, the distribution of Apo E genetypes, alleles frequencies, P and χ2 –values of HWE**

|  | **Total sample size** | | **Cases** | | | | | | | | | **Controls** | | | | | | | | |  |  |
| --- | --- | --- | --- | --- | --- | --- | --- | --- | --- | --- | --- | --- | --- | --- | --- | --- | --- | --- | --- | --- | --- | --- |
| **Authors** | Cases | Controls | E2/2 | E2/3 | E2/4 | E3/3 | E3/4 | E4/4 | E2 | E3 | E4 | E2/2 | E2/3 | E2/4 | E3/3 | E3/4 | E4/4 | E2 | E3 | E4 | ***χ*^2^** | ***P*_HWE_** ^§^ |
| Xie et al. [1] | 107 | 95 | 2 | 13 | 1 | 67 | 23 | 1 | 18 | 170 | 26 | 0 | 9 | 1 | 75 | 9 | 1 | 10 | 168 | 12 | 1.75 | 0.417 |
| Yuan et al. [2] | 103 | 100 | 0 | 6 | 2 | 88 | 7 | 0 | 8 | 189 | 9 | 0 | 13 | 1 | 80 | 6 | 0 | 14 | 179 | 7 | 1.17 | 0.557 |
| Zhang et al. [3] | 96 | 131 | 0 | 11 | 3 | 67 | 14 | 1 | 14 | 159 | 19 | 2 | 16 | 0 | 91 | 21 | 1 | 20 | 219 | 23 | 3.96 | 0.138 |
| Cao et al. [4] | 78 | 85 | 0 | 7 | 1 | 61 | 9 | 0 | 8 | 138 | 10 | 0 | 11 | 0 | 68 | 6 | 0 | 11 | 153 | 6 | 0.62 | 0.733 |
| Peng et al. [5] | 220 | 180 | 0 | 29 | 2 | 131 | 55 | 3 | 31 | 346 | 63 | 0 | 25 | 5 | 126 | 24 | 0 | 30 | 301 | 29 | 5.18 | 0.075 |
| Yan et al. [6] | 93 | 113 | 0 | 13 | 1 | 49 | 29 | 1 | 14 | 140 | 32 | 0 | 19 | 2 | 78 | 13 | 1 | 21 | 188 | 17 | 1.52 | 0.468 |
| Jin et al. [7] | 50 | 100 | 0 | 2 | 1 | 33 | 14 | 0 | 3 | 15 | 82 | 0 | 3 | 6 | 78 | 13 | 0 | 9 | 19 | 172 | 35.87 | 0.000 |
| Wu et al. [8] | 114 | 135 | 0 | 15 | 6 | 72 | 20 | 1 | 21 | 179 | 28 | 0 | 18 | 4 | 101 | 12 | 0 | 22 | 232 | 16 | 7.23 | 0.027 |
| Wang et al. [9] | 91 | 105 | 1 | 10 | 2 | 65 | 13 | 0 | 14 | 151 | 17 | 0 | 15 | 1 | 77 | 11 | 1 | 16 | 180 | 14 | 1.43 | 0.489 |
| Li et al. [10] | 95 | 46 | 1 | 6 | 3 | 61 | 21 | 3 | 11 | 149 | 30 | 1 | 4 | 1 | 34 | 6 | 0 | 7 | 78 | 7 | 3.36 | 0.186 |
| Yang et al. [11] | 204 | 136 | 1 | 18 | 0 | 153 | 24 | 8 | 20 | 348 | 40 | 1 | 12 | 1 | 106 | 15 | 1 | 15 | 239 | 18 | 1.26 | 0.533 |
| Zhang et al. [12] | 91 | 102 | 1 | 10 | 2 | 65 | 13 | 0 | 14 | 151 | 17 | 0 | 14 | 1 | 76 | 10 | 1 | 14 | 178 | 12 | 1.60 | 0.449 |
| Pan et al. [13] | 100 | 50 | 2 | 7 | 1 | 69 | 21 | 0 | 12 | 166 | 22 | 1 | 6 | 0 | 38 | 5 | 0 | 8 | 87 | 5 | 2.20 | 0.333 |
| Zhang et al. [14] | 71 | 69 | 0 | 4 | 5 | 46 | 16 | 0 | 9 | 112 | 21 | 0 | 8 | 1 | 53 | 7 | 0 | 9 | 121 | 8 | 0.97 | 0.616 |
| Zhang et al. [15] | 60 | 50 | 0 | 7 | 1 | 41 | 10 | 1 | 8 | 99 | 13 | 0 | 3 | 1 | 38 | 8 | 0 | 4 | 87 | 9 | 1.69 | 0.430 |
| Bai et al. [16] | 50 | 47 | 0 | 4 | 0 | 40 | 6 | 0 | 4 | 90 | 6 | 0 | 5 | 0 | 39 | 3 | 0 | 5 | 86 | 3 | 0.41 | 0.815 |
| Wang et al. [17] | 54 | 71 | 0 | 2 | 1 | 31 | 18 | 2 | 3 | 82 | 23 | 0 | 9 | 1 | 43 | 18 | 0 | 10 | 113 | 19 | 2.48 | 0.289 |
| Wu et al. [18] | 172 | 286 | 0 | 17 | 0 | 131 | 24 | 0 | 17 | 303 | 24 | 0 | 39 | 4 | 202 | 41 | 0 | 43 | 484 | 45 | 3.95 | 0.139 |
| Li et al. [19] | 125 | 116 | 1 | 17 | 4 | 64 | 38 | 1 | 23 | 183 | 44 | 0 | 20 | 1 | 81 | 13 | 1 | 21 | 195 | 16 | 1.79 | 0.409 |
| Cao et al. [20] | 37 | 72 | 0 | 5 | 2 | 28 | 2 | 0 | 7 | 63 | 4 | 0 | 9 | 4 | 56 | 3 | 0 | 13 | 124 | 7 | 20.79 | 0.000 |
| Peng et al. [21] | 150 | 157 | 1 | 21 | 1 | 93 | 30 | 4 | 24 | 237 | 39 | 1 | 13 | 1 | 122 | 18 | 2 | 16 | 175 | 23 | 2.80 | 0.247 |
| Liu et al. [22] | 120 | 121 | 1 | 18 | 5 | 75 | 16 | 5 | 25 | 184 | 31 | 0 | 21 | 2 | 88 | 8 | 2 | 23 | 205 | 14 | 9.10 | 0.011 |
| Zhang et al. [23] | 120 | 120 | 10 | 14 | 12 | 61 | 22 | 1 | 46 | 158 | 36 | 13 | 13 | 14 | 69 | 9 | 2 | 53 | 160 | 27 | 42.94 | 0.000 |
| Gu et al. [24] | 129 | 90 | 0 | 20 | 5 | 73 | 29 | 2 | 25 | 195 | 38 | 0 | 12 | 2 | 68 | 8 | 0 | 14 | 156 | 10 | 2.80 | 0.247 |
| Yang et al.-1 [25] | 84 | 39 | 1 | 6 | 3 | 54 | 19 | 1 | 11 | 133 | 24 | 0 | 5 | 4 | 22 | 8 | 0 | 9 | 57 | 12 | 6.89 | 0.032 |

**Table S2. Continue**

|  | **Total sample size** | | **Cases** | | | | | | | | | **Controls** | | | | | | | | |  |  |
| --- | --- | --- | --- | --- | --- | --- | --- | --- | --- | --- | --- | --- | --- | --- | --- | --- | --- | --- | --- | --- | --- | --- |
| **Authors** | Cases | Controls | E2/2 | E2/3 | E2/4 | E3/3 | E3/4 | E4/4 | E2 | E3 | E4 | E2/2 | E2/3 | E2/4 | E3/3 | E3/4 | E4/4 | E2 | E3 | E4 | ***χ*** | ***P*_HWE_**^§^ |
| Yang et al.-2 [25] | 40 | 31 | 2 | 0 | 0 | 17 | 21 | 0 | 4 | 55 | 21 | 3 | 8 | 4 | 13 | 3 | 0 | 18 | 37 | 7 | 3.74 | 0.154 |
| liao et al.[26] | 86 | 158 | 0 | 7 | 5 | 54 | 19 | 1 | 12 | 134 | 26 | 5 | 16 | 8 | 113 | 15 | 1 | 34 | 257 | 25 | 22.98 | 0.000 |
| Yin et al. [27] | 105 | 110 | 4 | 9 | 3 | 72 | 16 | 1 | 20 | 169 | 21 | 1 | 9 | 2 | 90 | 8 | 0 | 13 | 197 | 10 | 5.31 | 0.070 |
| Sun et al. [28] | 96 | 113 | 0 | 13 | 1 | 51 | 30 | 1 | 14 | 145 | 33 | 0 | 19 | 2 | 78 | 13 | 1 | 21 | 188 | 17 | 1.52 | 0.468 |
| liao et al. [29] | 90 | 90 | 0 | 5 | 6 | 58 | 19 | 2 | 11 | 140 | 29 | 0 | 10 | 1 | 71 | 8 | 0 | 11 | 160 | 9 | 0.93 | 0.628 |
| He et al. [30] | 60 | 104 | 1 | 2 | 4 | 40 | 8 | 5 | 8 | 90 | 22 | 4 | 9 | 5 | 77 | 6 | 3 | 22 | 169 | 17 | 30.11 | 0.000 |
| Ou et al. [31] | 200 | 100 | 1 | 28 | 1 | 98 | 67 | 5 | 31 | 291 | 78 | 0 | 17 | 1 | 66 | 14 | 2 | 18 | 163 | 19 | 2.95 | 0.229 |
| Feng et al. [32] | 68 | 70 | 1 | 8 | 3 | 43 | 13 | 0 | 13 | 107 | 16 | 0 | 10 | 1 | 52 | 7 | 0 | 11 | 121 | 8 | 0.91 | 0.634 |
| Xiao et al.-1 [33] | 257 | 224 | 0 | 31 | 2 | 188 | 32 | 4 | 33 | 439 | 42 | 4 | 25 | 2 | 162 | 29 | 2 | 35 | 378 | 35 | 6.31 | 0.043 |
| Xiao et al.-2 [33] | 257 | 127 | 0 | 31 | 2 | 188 | 32 | 4 | 33 | 439 | 42 | 2 | 16 | 1 | 90 | 16 | 2 | 21 | 212 | 21 | 3.56 | 0.169 |
| Pan et al. [34] | 98 | 87 | 1 | 14 | 1 | 51 | 31 | 0 | 17 | 147 | 32 | 0 | 14 | 1 | 60 | 11 | 1 | 15 | 145 | 14 | 1.24 | 0.538 |
| Zou et al. [35] | 89 | 43 | 3 | 6 | 3 | 54 | 22 | 1 | 15 | 136 | 27 | 3 | 8 | 4 | 20 | 8 | 0 | 18 | 56 | 12 | 3.74 | 0.154 |
| Wang et al. [36] | 201 | 360 | 0 | 28 | 2 | 118 | 52 | 1 | 30 | 316 | 56 | 3 | 46 | 1 | 263 | 45 | 2 | 53 | 617 | 50 | 2.68 | 0.262 |
| Liang et al. [37] | 133 | 122 | 2 | 30 | 4 | 82 | 13 | 2 | 38 | 207 | 21 | 0 | 23 | 2 | 79 | 16 | 2 | 25 | 197 | 22 | 2.91 | 0.233 |
| Xie et al. [38] | 78 | 100 | 0 | 11 | 3 | 45 | 15 | 4 | 14 | 116 | 26 | 0 | 7 | 1 | 86 | 6 | 0 | 8 | 185 | 7 | 2.19 | 0.335 |
| Ma et al. [39] | 88 | 75 | 0 | 18 | 11 | 47 | 8 | 4 | 29 | 120 | 27 | 0 | 9 | 0 | 55 | 11 | 0 | 9 | 130 | 11 | 1.78 | 0.411 |
| Zhao et al. [40] | 29 | 116 | 0 | 4 | 1 | 19 | 4 | 1 | 5 | 46 | 7 | 0 | 15 | 1 | 90 | 9 | 1 | 16 | 204 | 12 | 2.41 | 0.300 |
| Chu et al. [41] | 329 | 220 | 7 | 58 | 9 | 162 | 92 | 0 | 81 | 474 | 101 | 0 | 41 | 5 | 140 | 34 | 0 | 46 | 355 | 39 | 5.02 | 0.081 |
| Xu et al. [42] | 52 | 60 | 0 | 7 | 3 | 32 | 9 | 1 | 10 | 80 | 14 | 0 | 8 | 1 | 46 | 5 | 0 | 9 | 105 | 6 | 1.17 | 0.557 |
| Tang et al. [43] | 26 | 60 | 0 | 1 | 0 | 14 | 9 | 2 | 1 | 3 | 13 | 0 | 3 | 1 | 43 | 13 | 0 | 4 | 102 | 14 | 1.64 | 0.440 |
| Wang et al. [44] | 30 | 30 | 0 | 3 | 2 | 18 | 7 | 0 | 5 | 46 | 9 | 0 | 4 | 1 | 21 | 4 | 0 | 5 | 50 | 5 | 1.25 | 0.535 |
| Sun, et al. [45] | 50 | 156 | 0 | 7 | 2 | 31 | 9 | 1 | 9 | 78 | 13 | 0 | 21 | 3 | 118 | 14 | 0 | 24 | 271 | 17 | 3.62 | 0.164 |
| Ding et al. [46] | 52 | 110 | 0 | 6 | 3 | 40 | 3 | 0 | 9 | 89 | 6 | 0 | 11 | 5 | 67 | 27 | 0 | 16 | 172 | 32 | 6.32 | 0.042 |
| Yang et al. [47] | 80 | 60 | 0 | 12 | 3 | 45 | 18 | 2 | 15 | 120 | 25 | 0 | 8 | 2 | 44 | 5 | 1 | 10 | 101 | 9 | 4.75 | 0.093 |

**Table S2. Continue**

|  | **Total sample size** | | **Cases** | | | | | | | | | **Controls** | | | | | | | | |  |  |
| --- | --- | --- | --- | --- | --- | --- | --- | --- | --- | --- | --- | --- | --- | --- | --- | --- | --- | --- | --- | --- | --- | --- |
| **Authors** | Cases | Controls | E2/2 | E2/3 | E2/4 | E3/3 | E3/4 | E4/4 | E2 | E3 | E4 | E2/2 | E2/3 | E2/4 | E3/3 | E3/4 | E4/4 | E2 | E3 | E4 | ***χ*^2^** | ***P*_HWE_ ^§^** |
| Yuan, et al. [48] | 220 | 100 | 0 | 24 | 8 | 148 | 38 | 2 | 32 | 358 | 50 | 0 | 11 | 2 | 78 | 9 | 0 | 13 | 176 | 11 | 3.10 | 0.212 |
| Gao et al. [49] | 190 | 92 | 2 | 12 | 6 | 122 | 42 | 6 | 22 | 298 | 60 | 2 | 8 | 2 | 68 | 12 | 0 | 14 | 156 | 14 | 6.71 | 0.035 |
| Han et al. [50] | 118 | 123 | 5 | 16 | 8 | 56 | 17 | 16 | 34 | 145 | 57 | 8 | 11 | 7 | 79 | 11 | 7 | 34 | 180 | 32 | 43.66 | 0.000 |
| Huang et al. [51] | 94 | 100 | 5 | 3 | 6 | 47 | 29 | 4 | 19 | 126 | 43 | 12 | 19 | 9 | 49 | 11 | 0 | 52 | 128 | 20 | 15.80 | 0.000 |
| Guo et al. [52] | 75 | 40 | 0 | 5 | 4 | 38 | 26 | 2 | 9 | 107 | 34 | 1 | 4 | 1 | 27 | 7 | 0 | 7 | 65 | 8 | 2.65 | 0.266 |
| Li et al. [53] | 121 | 107 | 0 | 12 | 2 | 85 | 20 | 2 | 14 | 202 | 26 | 1 | 15 | 1 | 81 | 9 | 0 | 18 | 186 | 10 | 0.38 | 0.827 |
| Mo et al. [54] | 97 | 35 | 0 | 14 | 1 | 51 | 30 | 1 | 15 | 146 | 33 | 0 | 6 | 1 | 24 | 4 | 0 | 7 | 58 | 5 | 1.04 | 0.595 |
| Ma et al. [55] | 89 | 78 | 0 | 20 | 2 | 40 | 21 | 6 | 22 | 121 | 35 | 3 | 25 | 3 | 38 | 6 | 3 | 34 | 107 | 15 | 9.31 | 0.010 |
| Lei et al. [56] | 213 | 198 | 1 | 16 | 3 | 139 | 51 | 3 | 21 | 345 | 60 | 0 | 17 | 1 | 131 | 45 | 4 | 18 | 324 | 54 | 1.68 | 0.432 |
| Wang et al. [57] | 99 | 78 | 0 | 15 | 1 | 70 | 12 | 1 | 16 | 167 | 15 | 2 | 14 | 0 | 44 | 17 | 1 | 18 | 119 | 19 | 3.59 | 0.166 |
| Zhang et al. [58] | 60 | 50 | 10 | 0 | 0 | 23 | 20 | 7 | 20 | 66 | 34 | 6 | 2 | 0 | 30 | 7 | 7 | 12 | 67 | 21 | 52.58 | 0.000 |
| Wang et al. [59] | 50 | 50 | 0 | 9 | 0 | 27 | 14 | 0 | 9 | 77 | 14 | 0 | 8 | 1 | 27 | 14 | 0 | 9 | 76 | 15 | 2.53 | 0.282 |

§ P -value for Hardy-Weinberg equilibrium in controls

**REFERENCES**

1. Xie Y, Guo S, Wang Z, Yin Y (1989) Epsilon 4: a genetic factor susceptible to hypercholesterolemia. Zhonghua yi xue za zhi 69: 585-587.

2. Yuan R, Liang S, Mao Y (1998) The Study of Interrelationship between Apolipoprotein E Alleles Polymorphism and Coronary Heart Disease. Chinese journal of cardiology 3: 320-322.

3. Zhang Y, You K, Zhang L, Jiang Y, Xue H, et al. (1998) Effect of apolipoprotein E polymorphism on serum lipids, coronary heart disease, and carotid artery atherosclerosis. Chinese Journal of Cardiology 26: 443-447.

4. Cao W, Chen F, Teng L, Wang S, Fu S, et al. (1999) The relationship between apolipoprotein E gene polymorphism and coronary heart disease and arteriosclerotic cerebral infarction. Chinese Journal of Medical Genetics 16: 249-251.

5. Peng J, Gong W, Peng S, Wang J, Shi L, et al. (1999) Effection of Apolipoprotein E Gene Polymorphism on Plasm Lipid Level and Association with coronary heart disease. Chinese Journal of Arteriosclerosis 7: 307-310.

6. Yan S, Zhou X, Lin Q, Song Y (1999) Association of polymorphism of apolipoprotein E gene with coronary heart disease in Han Chinese. Chin Med J (Engl) 112: 224-227.

7. Jin W, Lu Y, Li W, Xu Y, Zheng M (1999) Apolipoprotein E Polymorphism in Acute Myocardial Infarction and Its Relation to Serum Lipids. Chinese Journal of Arteriosclerosis 7: 51-53.

8. Wu X, Long J, Yin S, Huang S (2000) The relations between apolipoprotein E polymorphism and diseases. Shanghai Journal of Medical Laboratory Sciences 13: 129-130.

9. Wang X, Gao H (2000) Cl inical study of the association between apoE polymorphism and lipid metabolism of patients with CHD. Journal of Clinical Cardiology 16: 440-442.

10. Li W, Huang D, Du X, Zhang W, Hu J (2000) Effect of Apolipoprotein E Polymorphisms on Serum Lipids and Relation to Coronary Stenosis. Chinese Journal of Arteriosclerosis 8: 54-57.

11. Yang Z, Zhu T, Ma G, Yin H, Qian W, et al. (2001) Apolipoprotein E polymorphism in the early onset of coronary heart disease. Chin Med J (Engl) 114: 983-985.

12. Zhang Z, Wang X, LI X, Zhang H, Gao H (2001) Clinical study of the association between apoe polymorphism and limid metabolism in patients with CHD. Acta Academiae Medicinae Shandong 39: 295-297.

13. Pan Y, Hu J, Li D, Pan Z, Wang Y (2001) Effects of ApoE polymorphism on plasma lipid levels and coronary disease. J Clin Intern Med 18: 267-269.

14. Zhang S, Cui Y (2001) Relationship between apolipoprotein E polymorphism and coronary heart disease. Chinese Journal of Practical Internal Medicine 21: 18-20.

15. Zhang G, Chen B, Jiang Z, Wen Q, Lu Y (2001) Effects of ApoE polymorphism on plasma lipid levels and coronary disease. Chin J Arterioscler 9: 310-312.

16. Bai X, Zhao M, Wang B, Guo R, Chen Y (2001) Dyslipidemia-related risk factors for myocardial infarction and polymorphism of ApoE gene among myocardial infarction patients and their siblings. Zhonghua yi xue za zhi 81: 340-343.

17. Wang G, Wang X, Yang C, Li X, Xiao B, et al. (2001) Relationship between apolipoprotein E gene polymorphism and longevity, lipids in Uygur Nationality. Chinese Journal of Gerontology 21: 325-327.

18. Wu JH, Lo SK, Wen MS, Kao JT (2002) Characterization of apolipoprotein E genetic variations in Taiwanese: association with coronary heart disease and plasma lipid levels. Hum Biol 74: 25-31.

19. Li W, Guan SM, Qi BL, Ke QM, Zhang HP (2003) The clinical significance of the detection of apolipoprotein E polymorphism for coronary heart disease in the elderly. population. Chin J Geriatr (Chin) 22: 214-217.

20. Cao L, Liu H, Du P, Fu G (2003) Correlation Between Apolipoprotein E Gene Polymorphism and Alzheimer and Coronary Heart Disease. Journal of Chinese Physician 5: 741-743.

21. Peng DQ, Zhao SP, Nie S, Li J (2003) Gene-gene interaction of PPARgamma and ApoE affects coronary heart disease risk. Int J Cardiol 92: 257-263.

22. Liu XC, Peng HY, Qin GF (2003) The relationship of ApoE polymorphisms with the serum concentration of ApoE in coronary heart disease. Shanghai J Med Lab Sci (Chin) 18: 36-39.

23. Zhang D, Wen S, Cui G (2003) A Study of the Relationship Between Apolipoprotein E Gene Polymorphism And CHD in Young People Qingdao Medical Journal 35: 84-85.

24. Gu W, Zhu J, Pan M, Yuan J, Gong Y, et al. (2003) Effects of angiotensinogen and apolipoprotein E gene polymorphisms on coronary heart disease. ACTA Academiae Medicinae Nantong 23: 394-395.

25. Yang S, He B, He Z, Zhang H, Hong X, et al. (2003) Apolipoprotein E Gene Polymorphisms and Risk for Coronary Artery Disease in Chinese Xinjiang Uygur and Han Population. Chinese Journal of Arteriosclerosis 11: 429-434.

26. Liao Q, Liang Z, Hu S, li l, LU X (2004) Zhuang people of Guangxi apolipoprotein E gene polymorphism and risk of coronary heart disease. Journal of Sichuan University (Medical Sciences Edition) 35: 285-286.

27. Yin Z, Li R, Zhao Y, Chang X, Wang H, et al. (2004) The study on the interrelation between polymorphism of apolipoprotein E and HsCRP in patients with coronary heart disease. Foreign Medical Sciences(section of Clinical Biochemistry and Laboratory Medicine) 25: 387-389.

28. Sun H, Wang B, Wang X (2004) Correlative Experiment of Apolipoprotein E Gene Polymorphism on Coronary Heart Disea se and It s Clinical Research. Journal of Beihua University (Natural Science) 5: 534-537.

29. Liao M, Jiang B, Xu C, Xu F, Huang X (2004) The study of relationship between apolipoprotein E polymorphism and coronary heart disease. Practical Preventive Medicine 11: 691-693.

30. He J, Gui J, Yu W, Chou D (2004) An Study on the Association of Apolipoprotein E Genetypes with Cerebral infarction and Myocardial infarction in the Urumqi Old Population. Journal of Chinese Physician: 49-51.

31. Ouyang T, Song JN, Miao Y, Lin Q, Niu XH, et al. (2005) Study on relationship between polymorphism of apolipoprotein E gene and syndromes of phlegm and blood stasis in patients with coronary heart disease. Zhong xi yi jie he xue bao = Journal of Chinese integrative medicine 3: 438-442.

32. Feng S, Wang H, Wang Q, Tian J (2005) Analysis of apolipoprotein E gene polymorphisms in elderly coronary heart disease. Geriatr Health Care (Chin) 11: 174-176.

33. Xiao ZJ, Zhao SP, Nie S, Tan LM, Jiang B, et al. (2005) The relationship between ApoE gene polymorphism and lipid parameters. Chinese Journal of Epidemiology 26: 533-536.

34. Pan JH, Xu ZQ, Hou ZL, Yu Z (2005) Significance of apolipoprotein E gene polymorphism in patients with coronary heart disease. Medicine and Pharmacy of Yunnan 26: 500-503.

35. Zou Y, Hong X, Hu D, Yang X, Jia X, et al. (2005) Relationships Between apolipoprotein E gene polymorphisms and coronary heart disease. Chinese Journal of Arteriosclerosis 13: 355-358.

36. Wang C, Zhou X, Ye S, Han D, Tan X, et al. (2006) Combined effects of apoE-CI-CII cluster and LDL-R gene polymorphisms on chromosome 19 and coronary artery disease risk. International Journal of Hygiene and Environmental Health 209: 265-273.

37. Liang Q, Yang X, Yang G, Cui J (2006) Relationship of Angiotensin-converting Enzyme, Angiotensinogen and Apolipoprotein E Gene Polymorphisms with Coronary Disease. Clinical Medical Journal of China 13: 722-724.

38. Xie L, Lai R, Li Q, S L, Z C, et al. (2006) DNA Sequencing research on apolipoprotein E polymorphism in Jiangsu with cardiovascular and cerebrovascular disorders. Acta Universitatis Medicinalis Nanjing(Natural Science) 26: 505-519.

39. Ma H, Li F, Guan L (2006) Association of ApoE gene polymorphism with coronary heart disease and lipid metabolism. Journal of Binzhou Medical University 29: 257-260.

40. Zhao X, Zhang Z, Jia M, Zhang Y (2006) Association of apolipoprotein E polymorphism with plasma lipids and atherosclerosis. Medical Journal of Chinese People’s Health 18: 88-94.

41. Chu Y, Chu Z, Zhu Y (2007) Research on the Relationship between Apolipoprotein E Gene Polymorphism and Early-noset Coronary Heart Disease. Progress in Modern Biomedieine 7: 244-246.

42. Xu Y, Shi Y (2007) Study on the relationship of apolipoprotein E gene polymorphism with coronary heart disease. Journal of Qiqihar Medical College 28: 2444-2445.

43. Tang L, Wang X, Yv K, Zhang H, Zheng J, et al. (2007) Correlative analysis of apolipoprotein B、E gene polymorphism and several common diseases in southern area of Zhejiang Province. Journal of Wenzhou Medical College 37: 14-17.

44. Wang X, Li Y, Zeng M, Ma J, Wang J (2007) Association Between Apolipoprotein E genotype and Coronary Heart Disease. Journal of Changsha Medical College 6: 8-10.

45. Sun Z, Li L, Ma QH (2008) Effects of ApoE polymorphism on plasma lipid levels and coronary heart disease. Medical Journal of Liaoning 22: 7-11.

46. Ding F, Liu H, Liu T (2008) Analysis of relationship between apolipoprotein E gene polymorphism and early onset of coronary heart disease. China Tropical Medicine 8: 41-42.

47. Yang L, li S, Qi F, Shi Y, Guo R, et al. (2008) Relationship between apoliprotein E gene polymorphisms in patients with coronary heart disease and serum lipids. China Journal of Modern Medicine 18: 364-369.

48. Yuan Z, Huang X, Tan G, Li J, Hu Z, et al. (2008) Detective analysis on polymorphism of apolipoprotein E gene in bloodstasis syndrome of coronary heart disease. Journal of Beijing University of Traditional Chinese Medicine 31: 830-834.

49. Gao C, Wang Y (2008) Relationship between Apolipoprotein E gene polymorphism and coronary artery disease. Clinical Focus 23: 1549-1550.

50. Shi YR, Zhou HY, Zhang Y (2009) Relationship of gene polymorphisms of fatty acid binding protein-2 and apolipoprotein E with coronary heart disease in type-2 diabetic patients. Journal of Xi'an Jiaotong University (Medical Sciences) 30: 85-88.

51. Huang Y, He Y (2009) The correlation of the polymorphism of apolipoprotein E and high sensitivity C-reactive protein for coronary heart disease. Chinese Journal of Gerontology 29: 2864-2866.

52. Guo J, JU J, Xu X, liu C (2009) Association of Polymorphism of Cystatin C Gene and Polymorphism of Apolipoprotein E Gene in Coronary Heart Disease. Journal of Modern Laboratory Medicine 24: 40-43.

53. Li H, Zhao S (2009) Interaction between ApoE and cholesterylester transferprotein polymorphism and effects on coronary heart disease. Clinical Focus 24: 2127-2130.

54. Mo H, Pan P, Huang Y, Hong Y, Wang Y (2010) Study on correlation between Coronary heart disease, phlegm and blood stasis syndrome and apolipoprotein E gene polymorphism. Journal of Guangzhou University of Traditional Chinese Medicine 27: 418-423.

55. Ma J, Zhang H, Wang W, Tian Q (2011) The Research of Apolipoprtein B，E and Angiotensinogen Gene Polymorophisms in Predisposition to CHD. China modern doctor 49: 4-6.

56. Lei Y, Zhang C, Wang X, Cao T, Lin S, et al. (2012) The relationship of ApoE gene polymorphism and coronary heart disease between Uighurs and Kazaks in Xinjiang China. Chinese Journal of Immunology 28: 807-809.

57. Wang S, Lei Y, Xie L, Liu Y (2012) ApoE gene Polymorphism in Xinjiang Uygur and Han Population with Coronary Heart Disease Journal of Xinjiang Medical University 35: 1643-1650.

58. Zhang F, Fan M, Liu Y, Liu W, Li L (2012) Research on the relationship between apolipoprotein E gene polymorphism and lipids in patients with coronary heart disease. Journal of Hainan Medical University 112: 1256-1258.

59. Wang Y, Wu C, Yao M, Zhang Y, Zheng L (2012) Study on the apoe gene polymorphism in Li populations patients with cardiovascular disease. Laboratory Medicine 27: 308-315.
